# The Government Stamp on Proprietary Medicines

**Published:** 1899-03-25

**Authors:** 


### THE GOVERNMENT STAMP ON PROPRIETARY MEDICINES.

We have often pointed out the evils connected with the use of the Government stamp on proprietary medicines, a use which undoubtedly gives them a fictitious value, and seems, in the eyes of the public, to afford a sanction for their use. A curious illustration of the extent to which this misconception may go was afforded during the discussion of a paper on "Warburg's Tincture" at a recent meeting of the New York County Medical Association, in the course of which Dr. Thomas Manley remarked that the genuine Warburg's Tincture was a proprietary medicine, and that its manufacture was entirely controlled by the British Government (!) This, we take it, is a fair illustration of the *suggestio falsi* which results from such drugs being, as the proprietors say, "protected by the Government stamp."

